# Assessing and Mitigating Local Vulnerabilities to Completeness of Global Polio Eradication

**DOI:** 10.1093/jpids/piab102

**Published:** 2021-11-03

**Authors:** Ananda S Bandyopadhyay, Walter A Orenstein

**Affiliations:** 1 Polio, Global Development Division, Bill & Melinda Gates Foundation, Seattle, Washington, USA; 2 Department of Medicine, Emory University, Atlanta, Georgia, USA

The battle to eradicate poliomyelitis is at a crossroads in 2021 with endemic wild-type poliovirus (WPV) transmission at historic lows in Pakistan and Afghanistan, whereas the emerging and ongoing outbreaks of circulating type 2 vaccine-derived polioviruses (cVDPV2) pose a serious challenge to the global program [1]. Two papers in this issue reporting studies in Chad and India illustrate both the risks of re-introduction of virus transmission in polio-free geographies as well as affordable mitigation options to protect the populations from paralytic disease [2, 3].

In a remarkable feat that upholds the principles of immunization and disease eradication, the Region of Africa was certified free from all 3 WPVs in August 2020 [4]. However, the expansion of cVDPV2 outbreaks which have affected at least 21 countries across the Region in recent times is a reminder that areas with persistently low immunization coverage are at risk of re-introduction of poliovirus transmission [5]. In this context, understanding the vulnerability of sub-populations to risk of transmission of different serotypes of polio is critically important. Gamougam et al [2] have surveyed a cohort of children from 1 to 5 years of age in Chad to measure their seroprevalence for antibodies against the 3 polio serotypes. They found 90.7%, 61.4%, and 86.2% of 236 evaluable children had detectable antibodies against types 1, 2, and 3, respectively. The low level of humoral antibodies against type 2 poliovirus indicates substantial population susceptibility and hence a risk of paralytic poliomyelitis in this sub-population given the backdrop of circulating type 2 poliovirus outbreaks in the region. Such low levels of protective antibodies also explain why cVDPV2 outbreaks occurred in Chad in 2019 and 2020 [6].

The Global Polio Eradication Initiative (GPEI) strategy to switch in 2016 from use of trivalent to bivalent Oral Polio Vaccine (OPV) was accompanied by a recommendation to include at least one dose of intramuscularly administered full-dose trivalent inactivated poliovirus vaccine (“IPV”) in essential immunization schedules to minimize the risks of paralytic disease from cVDPV2 and to enhance protective immunity against the other types of polioviruses. However, limited global manufacturing capacity resulting in interrupted supplies of IPV and sub-optimal routine immunization coverage in resource-constrained settings contributed to insufficient protection against type 2 poliovirus in the post-switch era.

Previous studies have established that 2 or more doses of fractional-inactivated poliovirus vaccine (f-IPV) can substitute for full doses of IPV to provide immunity in children depending on the age of administration [7, 8]. The study by Ahmad et al [3] focuses on the use of 2 doses of f-IPV, each containing one-fifth of the full dose of intramuscularly administered IPV, to induce immunity against type 2 poliovirus when primary immunity against types 1 and 3 is provided by Bivalent Oral Polio Vaccine (bOPV). Such schedules are aligned with the GPEI-supported policy to address supply and cost constraints of IPV in essential immunization schedules along with bOPV. This strategy has been adopted in India where f-IPV is administered intradermally to infants at 6 and 14 weeks of age per the Universal Immunization Program. However, an issue with adoption of intradermal vaccination is the injection technique using small syringes and needles designed for intradermal BCG immunization, something which many vaccinators are untrained for. Each of the 4 arms of the study comprised 200 Indian infants. The different arms included 2 different schedules of f-IPV doses (at 6 and 14 weeks and 10 and 14 weeks), an arm in which an alternative to the BCG needle/syringe, the West intradermal adapter from Helm Medical (at 6 and 14 weeks), was used, and a cohort that received a full dose of IPV administered intramuscularly at 14 weeks as comparator. The primary outcome measure was the seroconversion rate for type 2 with the intention of demonstrating non-inferiority of 2 doses of f-IPV compared with the full-dose IPV. Also assessed were immune responses to all 3 polio types at 14 and 18 weeks, as well as local and systemic adverse events using 7-day diary cards completed by parents.

The primary study objective was met, with observation of a significant improvement in the type 2 seroconversion rate following 2 doses of f-IPV at 6 and 14 weeks compared with a single full dose of IPV. Type 2 seroconversion was 85.8% (95% CI 80.1-90.0) 4 weeks after the second dose, and 67.9% (95% CI 60.4-74.6) 4 weeks after a single dose of IPV, a difference of 17.9% (*P* < .001), indicating in this study that 2 fractional doses at 6 and 14 weeks induced favorable immunogenicity compared to a single full dose at 14 weeks. There was also a significant difference in the median titer of antibodies against type 2, which was 57 (95% CI 45-72) for the f-IPV arm vs 18 (95% CI 14-22) for the IPV arm (*P* < .001). When the f-IPV schedule was 10 and 14 weeks, the seroconversion rate 4 weeks later was 77.0% (95% CI 70.5-82.5), a nonsignificant increase of 9.1% (*P* = .057) over a single full-dose IPV, but 8.8% less than the 6 and 14 weeks schedule (*P* = .028). The lower seroconversion rate with delayed administration of f-IPV at 10 and 14 weeks compared with that administered at 6 and 14 weeks is difficult to explain as it contradicts the understanding that maternally derived antibodies, which would be expected to be higher at 6 weeks than 10 weeks, interfere with IPV immunogenicity. The relative impact of longer interval between doses and other factors on immunogenicity compared with the impact from maternally derived antibodies are worth exploring further.

The use of the novel intradermal adapter was associated with similar seroconversion rates and titers of type 2 neutralizing antibodies, but with an improvement in the proportion of the vaccine dose actually delivered, assessed as wetness around the injection site. Other factors associated with use of the novel adapter showed minimal differences, notably bleb size and time taken for administration. These factors may be important as bleb size has previously been shown to be associated with the immunogenicity of intradermal f-IPV administration [9].

The authors make an interesting observation that 22 of the infants in the IPV group seroconverted against type 2 poliovirus before receiving their first dose of any type 2 containing vaccine. Investigations to determine why these participants seroconverted to type 2 poliovirus failed to find any definitive explanation; affected participants were distributed across the different study sites and there was no evidence of any localized VDPV2 circulation. Such a finding indicates the possibility of ongoing passive exposure in the community from either undetected VDPV2 circulation or the inadvertent or illicit use of type 2 containing OPV.

In the years to come, further exploration of alternative, IPV-only schedules with fewer doses along with outbreak response use of novel oral polio vaccines that are less likely to seed new emergences would be important to inform policies [8, 10]. The results of the 2 studies in Chad and India described here illustrate the importance of continuing to adequately vaccinate infants and children against type 2 poliovirus and monitor the impact of vaccination activities with serologic evaluations. Optimum use of more affordable options such as the f-IPV and other novel vaccine choices could allow a fairer, wider, and safer adoption of polio vaccine and vaccination choices in developing countries and provide further support for the global eradication of all forms of polioviruses.

## Note

***Potential conflicts of interest***. The authors declare no potential conflicts of interest. Both authors have submitted the ICMJE Form for Disclosure of Potential Conflicts of Interest. Conflicts that the editors consider relevant to the content of the manuscript have been disclosed.
